# Gut-muscle axis in sarcopenia: from mechanisms to microbiome-based therapies

**DOI:** 10.3389/fnut.2026.1846179

**Published:** 2026-08-17

**Authors:** Yikai Liu, Shuai Lu, Lei Li, Jiawei Yin, Pengli Han, Yejun Zha, Xieyuan Jiang

**Affiliations:** 1 Department of Orthopedics and Traumatology, Beijing Jishuitan Hospital, Capital Medical University, Beijing, China; 2 Beijing Research Institute of Traumatology and Orthopedics, Beijing, China; 3 Department of Adult Joint Reconstructive Surgery, Beijing Jishuitan Hospital, Capital Medical University, Beijing, China; 4 Pharmaceutical Department, Zhengzhou Central Hospital Affiliated to Zhengzhou University, Zhengzhou, China; 5 Department of Orthopedic Trauma, Zhengzhou Central Hospital Affiliated to Zhengzhou University, Zhengzhou, China

**Keywords:** sarcopenia, gut microbiota, inflammation, dietary intervention, probiotics

## Abstract

Sarcopenia is a disease characterized by reduced muscle mass and strength. In recent years, increasing evidence suggests that gut microbiota (GM) may play an important role in the pathogenesis of sarcopenia. GM is associated with muscle health by affecting muscle metabolism, regulating inflammatory responses, and promoting nutrient absorption. Despite recent rapid research progress, there are still many uncertainties regarding the causal relationship between GM and sarcopenia, key effector molecules, and individualized intervention strategies. We review the mechanisms of GM in the pathogenesis of sarcopenia with a focus on four interconnected pathways: chronic inflammation, nutrient absorption, amino acid metabolism, and GM-derived metabolites. This review aims to propose an integrated framework linking GM to muscle homeostasis to guide future research directions. Interventions targeting GM may offer a new approach for the treatment of sarcopenia, but more research is needed to clarify the causal relationship and optimize intervention measures.

## Introduction

Sarcopenia is an age-related condition characterized by generalized loss of skeletal muscle mass, strength, and physical function, which has a negative impact on the life quality and health status of elderly individuals. With the rapid aging of the world population, the prevalence of sarcopenia is increasing year by year, and about 10–16% of older adults are estimated to be impacted by this condition, making it as a major public health concern (1). The pathogenesis of sarcopenia is complex, involving multiple factors such as inflammation, oxidative stress, hormonal imbalance, and mitochondrial dysfunction. Recently, gut microbiota (GM), as a new regulatory factor, has attracted increasing attention (2, 3).

GM is the microbial community residing in the human gut, with a total count over 10^14^ and a gene count at least 100 times that of the human genome (4). These organisms are bacteria, fungi, viruses, and archaea, and bacteria is the dominant group, including phyla such as *Firmicutes, Bacteroidetes, Actinobacteria*, and *Proteobacteria*. GM participates in the host' s nutritional metabolism and plays additional important roles in human health by promoting food digestion and nutrients absorption. For instance, certain GM could

ferment dietary fiber to produce short-chain fatty acids (SCFAs), such as acetic acid, propionic acid, and butyric acid. These SCFAs are often discussed collectively, but they exert distinct effects on muscle homeostasis: acetate enhances mitochondrial function and glucose metabolism, butyrate suppresses inflammation and autophagy to preserve muscle mass, while propionate plays complex roles that may impair myogenic differentiation at high concentrations (5). Collectively, SCFAs provide energy for the host but also regulate intestinal barrier function and immune response (6, 7). Moreover, GM plays a pivotal role in host immune regulation. It can stimulate the development of the host immune system, enhance the intestinal mucosal barrier function, resist pathogen invasion, and maintain the balance of the immune system, thereby preventing inflammatory diseases (8).

Studies have shown that muscle mass and strength are strongly associated with the composition of GM (9–11). For instance, recent systematic reviews have reported a significant association between reduced GM diversity and the occurrence of sarcopenia, and the reduction of some specific bacteria, such as *Prevotella* and *Faecalibacterium* might be associated with muscle loss (12, 13). Moreover, in patients with chronic diseases such as heart failure and chronic kidney disease, changes in the GM are also closely associated with sarcopenia, which further supporting the concept of the gut-muscle axis (14, 15). Interestingly, diurnal temperature range in humans is positively associated with sarcopenia risk, and in mice, fluctuated temperature triggers muscle loss through dysregulating GM (e.g., reducing *Eubacterium*) and increasing circulating aminoadipic acid, which impairs mitochondrial mitophagy (16).

Mechanistically, specific GM facilitate muscle protein synthesis through the production of SCFAs, the reduction of low-grade inflammation, and the enhanced utilization of amino acids, thereby influencing muscle mass (17). In this context, dysbiosis of the GM may lead to chronic low-grade inflammation, which is considered a potential mechanism underlying the development of sarcopenia. Furthermore, metabolites of the GM, such as SCFAs and certain amino acids, may play a crucial role in maintaining muscle health. For example, research has found that the GM can indirectly regulate the physiological functions of muscles by affecting the host's metabolic state and immune response. Specifically, metabolites produced by GM may promote the maintenance of muscle mass by regulating energy metabolism and improving insulin sensitivity (18). In addition to these mechanisms, numerous probiotics have demonstrated efficacy against sarcopenia (19–21). These findings emphasize the important role of the GM in sarcopenia and suggest that regulating the GM may be an effective strategy for the prevention and treatment of sarcopenia (3). As individuals age, the composition of the GM changes, diversity decreases, and beneficial bacteria diminish, which may affect the dynamic balance of skeletal muscle through the “gut-muscle axis” and lead to the occurrence and progression of sarcopenia.

In summary, GM plays a complex and crucial role in the pathogenesis of sarcopenia. Future research should further explore how GM affects muscle health through various mechanisms. This will not only deepen our understanding of sarcopenia but also potentially provide insights for developing new prevention and treatment strategies. By regulating GM, we may be able to open up new avenues for improving muscle health and overall quality of life in the elderly.

### Diagnosis of sarcopenia

**Figure 1:**
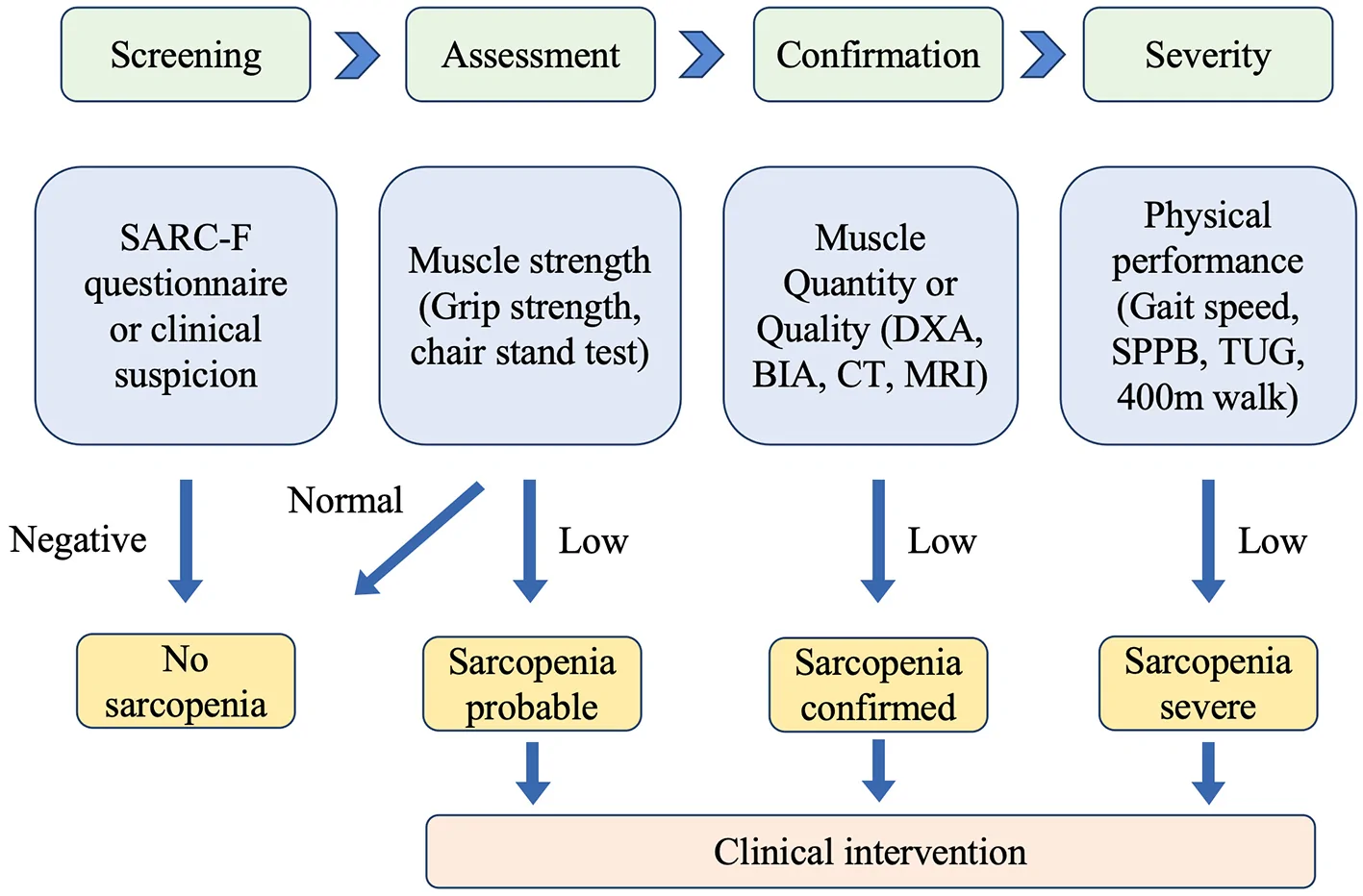
The diagnosis procedure of sarcopenia according to the EWGSOP2. BIA: bioelectrical impedance analysis; DXA: dual-energy X-ray absorptiometry; SPPB: Short Physical Performance Battery; TUG: Timed-up-and-go test.

Sarcopenia is a syndrome characterized by progressive and generalized decline in skeletal muscle mass and strength, closely related to aging and also influenced by various diseases (1). Currently, diagnosis of sarcopenia is mainly based on certain manifestations such as decreased muscle mass, reduced muscle strength, and limited physical function. In 2019 and 2020, diagnosis of sarcopenia was further revised by the European Working Group on Sarcopenia in Older People 2 (EWGSOP2) and the Asian Working Group on Sarcopenia (AWGS) (22, 23). The flow of the diagnostic procedure is detailed in Figure 1, and recent cut-off points for muscle mass, strength, and physical performance of EWGSOP2 and AWGS criteria are listed in the Table 1. Importantly, the diagnostic criteria (EWGSOP2 vs. AWGS) differ in their primary diagnostic parameters (muscle strength priority vs. muscle mass emphasis), measurement tools (DXA vs. BIA), cutoff values (e.g., gait speed < 1.0 m/s in AWGS vs. ≤ 0.8 m/s in EWGSOP2), and target populations (European vs. Asian). Due to the slight difference between these two criteria, the same individual may be classified as having sarcopenia under one criterion but not the other. The use of different sarcopenia diagnostic criteria across GM-related studies may influence findings, as this variation can lead to case-control misclassification and introduce confounding due to population stratification (e.g., differences in race, diet, and genetics between European and Asian populations). Therefore, when comparing GM-sarcopenia findings across studies, the diagnostic criteria applied should be explicitly considered, and subgroup analyses stratified by criteria are recommended.

**Table 1:** EWGSOP2 sarcopenia cut-off points.

Tests	EWGSOP2 Cut–off points	AWGS Cut–off points
Grip strength	< 27 kg for men, and < 16 kg for women	< 28 kg for men, and < 18 kg for women
Chair stand	>15 s for five rises	>12 s for five rises
ASM	< 20 kg for men, and < 15 kg for women	–
ASM/Height^2^	< 7.0 kg/m^2^ for men, and < 5.5 kg/m^2^ for women	< 7.0 kg/m^2^ for men; for women, < 5.4 kg/m^2^ using DXA or < 5.7 kg/m^2^ using BIA.
Gait speed	≤ 0.8 m/s	≤ 1.0 m/s
SPPB	≤ 8 point score	≤ 9 point score
TUG	≥20 s	–
400m walk test	Non–completion or ≥6 min for completion	–

### Pathophysiology of sarcopenia

The pathophysiology in development of sarcopenia is complex. Inflammation plays a significant role in this process. Pro-inflammatory cytokines such as tumor necrosis factor-α (TNF-α) and interleukin-6 (IL-6) are elevated in chronic inflammatory diseases, and inhibit muscle protein synthesis and enhance protein degradation, ultimately leading to muscle wasting (24). Additionally, factors such as neuromuscular junction dysfunction, mitochondrial dysfunction (25), and hormone imbalance also contribute to this process. For example, a decline in levels of hormones, such as growth hormone and insulin-like growth factor-1 (IGF-1), can impact muscle development and repair (26, 27). Furthermore, dysfunctional mitochondria in muscle cause aberrant energetics and contributes to muscle functional decline (28).

There are many biological factors associated with sarcopenia, such as those involving inflammation, metabolism, and muscle growth signaling, which help explain why muscles weaken with age and how to measure this deterioration. Among these, the serum creatinine-to-cystatin C ratio (Cr/CysC) has the best diagnostic ability and is the most common biomarker for sarcopenia (29). Conversely, the inflammatory biomarkers are the most common ones, which are found in higher concentrations among those affected by sarcopenia; notably, their levels of expression change after treatment (29). A systematic review and meta-analysis of 80 studies found that frailty and sarcopenia in elderly people are associated with similar alterations in metabolic, inflammatory, and hematologic biomarkers including albumin, hemoglobin, and IL-6, thus linking the biology of these 2 conditions (30). Beyond inflammation, myostatin, a TGF-β family member primarily expressed in skeletal muscle, acts as a negative regulator of muscle growth. It has been reported to be upregulated at the gene and protein levels with aging, with higher levels detected in older men when compared to younger men (31). A recent study found that mitochondrial calcium uniporter regulator 1 (MCUR1) is downregulated in aged human muscle, impairing mitochondrial calcium uptake and contributing to sarcopenia, and it identified oleuropein as a natural compound that activates MCU to restore mitochondrial function, improve endurance, and reduce fatigue in mice (28). Moreover, human sarcopenia may involve a dysfunction in anabolic signaling pathways (involving GHR, IGF-1, and Akt), with TNF-α, SOCS-3, and myostatin may be mediators in this impairment (32).

### Risk factors of sarcopenia

The occurrence and progression of sarcopenia are influenced by various risk factors, including GM. Age is a significant risk factor. As individuals age, the structure and function of the GM undergo changes (33). Higher body mass index was associated with an increased risk of sarcopenia (34, 35). Dietary habits have a notable impact on both the GM and sarcopenia. Malnutrition is one of the risk factors of sarcopenia, as sufficient intake of protein is essential for muscle growth (36). A diet high in sugar and fat and low in dietary fiber can lead to GM imbalance, elevate the risk of chronic inflammation and reduce the muscle integrity in rats (37).

Chronic diseases such as diabetes and cardiovascular disease are also important risk factors for intestinal dysbiosis and sarcopenia (38). Patients with diabetes often suffer from intestinal dysbiosis, characterized by a decrease in beneficial bacteria and an increase in harmful bacteria. Meanwhile, metabolic abnormalities such as insulin resistance can also affect muscle metabolism, increasing the risk of sarcopenia (39). Sarcopenia was related to higher cardiovascular disease risk among aged 40 and older Chinese adults (40). Some other chronic diseases, such as hypertension, chronic lung diseases, heart disease, liver cirrhosis (41), psychiatric disease and arthritis, are also associated with sarcopenia (42). In addition, drug use (such as antibiotics, proton pump inhibitors, etc.) and lifestyle (such as lack of exercise, smoking, excessive alcohol consumption, extreme sleep duration, etc.) may also indirectly affect the occurrence and development of sarcopenia (1, 42, 43).

### The evidence of interaction between GM and sarcopenia

#### Human observational evidence

In clinical research, studies have found that β-diversity was significantly associated with prevalent sarcopenia, indicating a negative correlation between GM diversity and muscle mass (44). Sarcopenia patients often show enrichment of certain harmful bacteria (e.g., *Enterobacteriaceae*) and a marked reduction in beneficial bacteria (e.g., *Bifidobacterium* and *Bacteroides*), with this change closely linked to muscle mass decline (45). Specific taxa elevated in sarcopenic individuals include *Desulfovibrio piger, Clostridium symbiosum, Hungatella effluvii, Bacteroides fluxus, Absiella innocuum, Coprobacter secundus*, and *Clostridium citroniae* (44). There are also limitations to these studies. For example, cross-sectional designs cannot establish causality or directionality. Associations may be confounded by age, diet, medication, and comorbidities. Small sample sizes and heterogeneous diagnostic criteria for sarcopenia also limit generalizability.

#### Animal and germ-free mouse evidence

Animal experiments have provided initial evidence linking GM to muscle mass regulation. Studies in aging mice have shown that GM composition changes correlate with declining muscle function (17, 46). In germ-free mouse models, the absence of GM leads to altered muscle characteristics, affecting muscle mass and strength (2, 9). Moreover, FMT studies in animals provide strong causal evidence, as transferring GM from sarcopenic donors to germ-free or conventional mice recapitulates muscle phenotypes (12, 47). However, animal findings may not fully translate to humans due to differences in gut architecture, diet, and immune systems. Generalizability is also constrained by short observation periods and controlled housing conditions that do not mimic human environmental complexity.

#### Causal inference evidence

Emerging evidence from Mendelian randomization studies supports a causal role of GM in sarcopenia. A study identified 21 GM-derived metabolites potentially associated with sarcopenia risk using two-sample Mendelian randomization (13). Another bidirectional Mendelian randomization analysis suggested that the GM may mediate the causal effect of inflammatory bowel disease on sarcopenia-related traits (48). Moreover, a meta-analysis synthesized data from 32 studies totaling 10,781 participants, confirming that individuals with sarcopenia have significantly lower GM diversity and distinct compositional features (49). Another 2025 meta-analysis of 10 studies (421 cases, 1,642 controls) corroborated these findings (50). These findings provide increasing support for a causal relationship, although large-scale prospective human trials are still needed.

#### Clinical intervention evidence

Interventions targeting GM have shown potential for improving muscle mass and function. Multiple studies report that certain probiotics and their beneficial metabolites (e.g., SCFAs) increase muscle synthesis markers, promoting muscle growth and recovery (19, 51). Short-term probiotics supplementation can improve muscle strength in elderly patients, suggesting a possible preventive and therapeutic strategy for sarcopenia (52). Therefore, interventions targeting GM not only offer a new perspective on the pathological mechanisms of sarcopenia but also provide potential directions for its treatment. However, most intervention studies are small, short-term, and use heterogeneous probiotic strains, doses, and formulations. The observed effects may be modest and not clinically meaningful. Generalizability is limited by age, baseline GM composition, and geographical differences in diet and lifestyle.

Collectively, animal models, observational studies, and preliminary clinical trials suggest a role of GM in sarcopenia. Future well-controlled randomized trials are needed to establish causality and therapeutic efficacy in humans.

### Changes of GM in sarcopenia

The association between GM and sarcopenia exhibits certain characteristics across different populations. In the elderly, age-related changes in GM are closely associated with the occurrence of sarcopenia. As individuals age, the diversity of GM decreases, beneficial bacteria diminish, and levels of chronic inflammation rise. These factors collectively increase the risk of sarcopenia (33, 53). For example, a study of elderly people living in the community found that those with frailty and sarcopenia had higher blood levels of aspartic acid, lower levels of threonine and macrophage inflammatory protein 1α, more of the gut microbes *Oscillospira* and *Ruminococcus*, and less *Barnesiellaceae* and *Christensenellaceae* (54). Another study of 88 older adults found that those with low muscle mass exhibited reduced GM diversity, a lower *Firmicutes*/*Bacteroidetes* ratio, decreased butyrate-producing bacteria (*Marvinbryantia*), and significantly lower fecal butyrate levels, which correlated with skeletal muscle mass index (55).

Among patients with chronic diseases, such as those with chronic liver disease and cancer, the coexistence of GM imbalance and sarcopenia is relatively common (56, 57). Taking patients with chronic liver disease as an example, due to factors such as impaired liver function and nutritional absorption disorders, intestinal flora imbalance often occurs, and the incidence of sarcopenia is also high. Studies have shown that the relative abundance of certain bacterial groups such as *Escherichia coli* and *Bacteroides* in the intestines of patients with cirrhosis is closely related to muscle mass index, suggesting that GM imbalance may play an important role in the occurrence of sarcopenia associated with chronic liver disease (58, 59).

### How does GM affect the pathogenesis of sarcopenia

The mechanisms linking GM to sarcopenia can be categorized by the strength of current evidence. Direct evidence (human studies, animal FMT, or mechanistic experiments) supports pathways involving SCFAs/mTOR signaling, chronic inflammation/NF-κB, and BCAA metabolism. Other pathways including bile acids, tryptophan metabolism, AMPK, and mitochondrial function, are supported primarily by indirect evidence and remain speculative until further validation.

#### GM and chronic inflammation

The GM regulates inflammatory responses through multiple mechanisms, including modulating the production of metabolites, regulating the activity of immune cells and the secretion of anti-inflammatory cytokines, and maintaining the intestinal barrier. SCFAs and other gut microbial metabolites can inhibit inflammatory responses (60, 61). One study demonstrated that SCFAs bind to GPR43 to promote resolution of inflammatory responses (61). This was supported by another study, which found that SCFAs inhibit inflammation by activating GPR41 and GPR43 on intestinal epithelial cells and triggering MAPK signaling pathway (62). Certain strains of probiotics, such as *Lactobacillus rhamnosus* HDB1258 could enhance the immune response by enhancing the phagocytic ability of macrophages and the cytotoxicity of natural killer (NK) cells (63). In addition, an 8-week trial in 121 healthy adults found that *Lacticaseibacillus rhamnosus* LM1019 significantly enhanced natural killer cell cytotoxicity in a subgroup aged ≥40 years with specific baseline immune and lipid profiles, suggesting selective immunomodulatory benefits (64). Moreover, the healthy GM prevent the infiltration of harmful bacteria and their metabolites into the blood circulation by maintaining the integrity of the intestinal barrier, thereby reducing the occurrence of systemic inflammation (65). Therefore, maintaining the balance of the GM is crucial for preventing chronic low-grade inflammation.

Dysbiosis of GM has been widely recognized as closely associated with the occurrence of various chronic diseases, especially chronic low-grade inflammation (66). Studies have shown that the diversity and composition of GM play a significant role in regulating chronic inflammation. For instance, dysbiosis of specific gut microbial communities can lead to excessive release of inflammatory factors such as IL-6 and tumor necrosis factor-α TNF-α (67). Inflammation plays a crucial role in the association between GM and sarcopenia (68). When GM is dysbiotic, the gut barrier function is compromised, allowing bacteria and their products, such as lipopolysaccharide (LPS), to translocate into the blood circulation, activating the immune system and triggering systemic chronic inflammation (69). Higher concentrations of inflammatory markers in the blood are linked to reduced skeletal muscle strength and mass (24). These cytokines can directly act on muscle tissue, inhibiting signaling pathways related to muscle protein synthesis, such as the mammalian target of rapamycin (mTOR) signaling pathway, while promoting the expression of genes related to protein degradation, leading to muscle atrophy (70). As people age, the diversity of GM tends to decrease, leading to a reduction in beneficial bacteria and an increase in pathogenic bacteria, thereby triggering chronic low-grade inflammation (68).

In addition to the harmful effects of inflammatory factors on muscles, chronic inflammation can also reduce muscle mass and function through various mechanisms. For instance, inflammatory response can indirectly affect muscle metabolism by influencing insulin sensitivity. One study revealed that chronic inflammation caused by obesity could reduce muscle glucose uptake and inducing insulin resistance by inhibiting the expression and translocation of glucose transporter type 4 (GLUT4) (71). Chronic inflammation can lead to insulin resistance, reducing muscle sensitivity to insulin, glucose uptake and utilization, and energy metabolism disorders, further exacerbating muscle functional decline (72). Moreover, mitochondrial function in skeletal myocytes is widely recognized as a primary driver of age-related muscle degeneration (73). One study demonstrated that LONP1, a mitochondrial protease, preserved muscle mass and strength during disuse by preventing the accumulation of mitochondrial proteins that would otherwise trigger autophagy-driven muscle loss (74). Prolonged low-grade inflammation could induce mitochondrial dysfunction (75). In turns, mitochondrial dysfunction triggers inflammation through the release of damage associated molecular patterns (DAMPs, such as mtDNA) and activation of inflammasomes (76). Collectively, the homeostasis of GM is beneficial for reducing chronic inflammation, thereby promoting muscle health.

#### GM and nutrient absorption

GM plays a crucial role in the digestion and absorption of nutrients. Studies have shown that gut microorganisms directly affect the absorption efficiency and metabolism of nutrients by influencing the function of intestinal epithelial cells. For instance, GM can produce SCFAs by decomposing dietary fiber. These SCFAs can not only be directly absorbed by intestinal cells but also regulate the intestinal microenvironment, enhancing the absorption capacity of other nutrients (77). Furthermore, GM significantly influences the metabolism of certain vitamins and minerals. GM could synthesize essential nutrients, including vitamins K and E, as well as antioxidants (78). By optimizing the composition of GM, nutrient absorption can be improved, thereby influencing overall metabolic health and reducing the risk of metabolic diseases. GM functionally interacts with key apical anion transporters—DRA (SLC26A3), PAT1 (SLC26A6), and CFTR (ABCC7)—thereby regulating intestinal epithelial transport activity (79). Moreover, obese patients often exhibit abnormalities in the structure of their GM, which may be associated with reduced nutrient absorption and metabolic efficiency. Obesity-related GM can alter the expression of nutrient transporters in intestinal epithelial cells, thereby affecting energy balance and fat storage (80). Therefore, regulating the composition and function of GM may be an important strategy to improve nutrient absorption.

#### GM and amino acids

GM plays a crucial role in the metabolism and bioavailability of amino acids, the building blocks of muscle protein. GM can synthesize essential amino acids, convert dietary proteins into bioactive compounds, and influence the absorption of amino acids from the gut. Dysbiosis can impair these functions, potentially limiting the supply of essential amino acids, particularly branched-chain amino acids (BCAAs: leucine, isoleucine, and valine). A metabolomics study identified specific GM and their genes responsible for this utilization (81). These metabolic capacities vary significantly across phyla: for example, L-asparaginase is ubiquitous in *Bacteroidota* and *Pseudomonadota*, while aromatic-amino acid aminotransferase and arginine deiminase are predominantly found in *Firmicutes* and *Actinomycetota*, and BCAA aminotransferase and proline racemase show exclusive or prevalent distribution in specific phyla (81). These differences indicate the different functions of GM in muscle synthesis.

BCAAs are key modulators of protein synthesis, metabolism, appetite, and longevity. (82). One study reported that leucine could stimulate muscle protein synthesis by insulin-dependent and –independent pathways, promote glucose disposal, and inhibit muscle proteolysis, thereby helping preserve muscle mass and function in older adults (83). A multi-ethnic study on 283 adults found that *Prevotella copri* abundance and BCAA levels were significantly lower in sarcopenic individuals, and *in vivo* experiments confirmed that *P. copri* supplementation in mice increased blood BCAAs levels and improved muscle mass and function, suggesting its therapeutic potential for sarcopenia (84). Another multi-omics study revealed impaired BCAAs catabolism as a major driver of sarcopenia, demonstrating that impaired BCAAs breakdown leads to muscle loss via dysregulated mTOR signaling, and that pharmacological (via BT2) or genetic enhancement of this pathway protects against sarcopenia in mouse models, highlighting BCAAs catabolism as a potential therapeutic target (85). Furthermore, a large community-based study of over 100,000 UK adults found that circulating BCAAs levels were positively associated with muscle mass and strength, with valine linked to a 47% reduced sarcopenia risk, which indicates that BCAAs may have potential efficacy in maintaining muscle health (86). Another study found that *Parabacteroides merdae* and its BCAAs-derived metabolites (isovalerate, 2-methylbutyrate, isobutyrate) delayed aging in mice by reducing oxidative stress and inflammation, improving muscle function and glucose regulation, and increasing *Bifidobacterium pseudolongum*, suggesting that targeting BCAAs-metabolizing gut microbes may be a novel anti-aging strategy (87).

However, conflicting evidence exists and must be carefully interpreted. A 12-month randomized controlled trial in 150 cirrhotic patients found that daily BCAAs supplementation did not significantly improve grip strength, upper limb lean mass, or other sarcopenia measures compared to an equicaloric whey protein control, indicating no added benefit over standard protein supplementation for sarcopenia in cirrhosis (88). Importantly, circulating BCAA levels (endogenous), BCAA supplementation (exogenous intervention), and enhanced BCAA catabolism (pathway modulation) are not equivalent concepts. Although BCAAs is a promising therapeutic target of sarcopenia, the intervention effect of BCAAs may depend on the underlying disease status, metabolic background of the body, and the endogenous utilization efficiency of BCAAs. Elevated circulating BCAAs may reflect impaired catabolism rather than sufficient availability, whereas BCAA supplementation aims to increase substrate supply, and genetic/pharmacological enhancement of catabolism promotes BCAA breakdown. Furthermore, BCAA-related effects are highly context-dependent. The efficacy of BCAA interventions may vary substantially across different disease conditions, including cirrhosis, chronic kidney disease, diabetes, obesity, and healthy aging. Factors such as underlying metabolic status, insulin resistance, inflammation, and GM composition could influence BCAA utilization and outcomes. Therefore, while BCAAs remain a promising therapeutic target for sarcopenia, future studies should consider disease-specific contexts, measure both circulating levels and catabolic capacity, and avoid generalizing findings from one population to another.

#### GM and metabolites

GM metabolites, particularly SCFAs derived from fiber fermentation, are key mediators in the development of sarcopenia. SCFAs serve as energy substrate for intestinal epithelial cells while also playing essential roles in antioxidant and the maintenance of GM homeostasis (89). Probiotics prevent muscle loss and maintain muscle function by enhancing SCFAs production. A study of 482 Chinese menopausal women integrated metagenomic, metabolomic, and Mendelian randomization analyses to demonstrate that gut microbial butyrate synthesis capacity has a causal positive effect on skeletal muscle mass, mediated by butyrate-producing species like *Faecalibacterium prausnitzii* and *Butyricimonas virosa* (90). One study demonstrated that SCFAs supplementation for 3 months in aged sarcopenic mice improved gut barrier function, reduced inflammation, and enhanced muscle mass, strength, and endurance by modulating AMPK, insulin, and mTOR signaling pathways, suggesting SCFAs as a potential therapeutic strategy for sarcopenia (17). These SCFAs not only directly affect the metabolism of muscle cells but also indirectly promote muscle synthesis and reduce muscle loss by regulating immune responses and inflammatory states (91). They support the maintenance of muscle mass by enhancing intestinal barrier function and reducing systemic inflammation levels. Specifically, acetate promotes mitochondrial and glucose metabolism; butyrate exerts anti-inflammatory and anti-autophagic effects to preserve muscle; propionate has context-dependent roles, aiding metabolism but inhibiting myogenesis at high levels (5). Importantly, these three SCFAs should not be considered interchangeable, as each acts through distinct molecular pathways. For example, butyrate can protect muscles by suppressing the activation of the nuclear factor-kappa B (NF-κB) signaling pathway, thereby reducing the expression of pro-inflammatory cytokines (92).

In addition to SCFAs, GM can also metabolize and produce other bioactive substances, such as bile acids (93, 94), indoles, and their derivatives, which may also be associated with sarcopenia. Bile acids not only participate in fat digestion and absorption but also regulate energy metabolism, inflammatory response, and intestinal barrier function by binding to specific receptors (93, 94). Studies have found that bile acid metabolism may be altered in patients with sarcopenia, with abnormal levels of certain bile acids affecting muscle metabolism and function. Dysregulation of bile acid signaling via FXR and TGR5 in skeletal muscle contributes to sarcopenia by impairing mitochondrial function, glucose uptake, and protein turnover through altered mTOR signaling and autophagy (95). In older adults, sarcopenia is linked to distinct changes in GM composition and tryptophan metabolism. Specifically, GM can accelerate muscle wasting by shifting tryptophan metabolism toward pro-inflammatory pathways, including kynurenine, serotonin, and indole, that promote inflammation in both the gut and muscles (96). Another study demonstrated that indoxyl sulfate, a protein-bound uremic toxin, accelerates skeletal muscle atrophy in chronic kidney disease by inducing oxidative stress, inflammatory cytokines, and the muscle atrophy-related genes myostatin and atrogin-1, as evidenced in both C2C12 myoblast cells and half-nephrectomized mice (97).

### Potential solutions for treating sarcopenia by intervening in GM

#### Probiotics

Probiotics and prebiotics also showed some promise in sarcopenia treatment (19–21). Probiotics are living microorganism with positive effect on the host by modulating GM. It has been confirmed that probiotics promote the abundance of beneficial bacteria, inhibit harmful bacteria colonization, thereby optimizing GM balance, and improve gut barrier function while reducing inflammatory responses (98, 99). Probiotics promote muscle health through different pathways, which results in an improvement of muscle mass and function as well as of sarcopenia symptoms. Both *in vivo* and *in vitro* evidence confirms that beneficial bacteria like *Lactobacillus* and *Bifidobacterium* enhance the systemic availability of amino acids and promote an anti-inflammatory environment in the intestinal lumen (100), and shows positive effects in improving muscle health in the elderly individuals (19, 20, 101–104). Notably, strains such as *Lactobacillus rhamnosus* and *Lactobacillus plantarum* were among the most frequently investigated and potentially promising strains for improving muscle quality and function. However, it is important to note that probiotic effects are strain-specific and should not be generalized at the genus or species level.

For instance, supplementing aging mice with *Limosilactobacillus reuteri ATG-F4* can restore the number of beneficial bacteria in the GM, reduce the production of inflammatory factors, and enhance muscle mass and exercise capacity (105). A six-week study in mice found that *Lactobacillus plantarum* TWK10 supplementation dose-dependently improved exercise performance and reduced fatigue by increasing grip strength, endurance time, relative muscle mass, and type I muscle fibers while decreasing post-exercise fatigue markers, indicating its feasibility to be used as an ergogenic resource (106). Furthermore, a double-blind trial of 55 elderly individuals with mild frailty found that high-dose supplementation of *Lactobacillus plantarum* TWK10 for over 6 weeks significantly improved muscle strength and endurance, reducing symptoms of sarcopenia and physical frailty (101). Another study found that *Lactobacillus plantarum* PL-02 supplementation plus resistance training for 4 weeks synergistically enhanced muscle mass and exercise performance and ameliorated fatigue markers in rats, which was superior to any of the two interventions alone (107). This study was strengthened by another study, which reported that 4 weeks of *Lactobacillus plantarum* PL-02 supplementation in mice significantly improved exercise performance and reduced fatigue by increasing muscle mass, strength, glycogen storage, and lowering post-exercise fatigue markers (107).

In addition, 12 weeks of *Lactobacillus casei* Shirota supplementation in aged SAMP8 mice mitigated age-related muscle decline by modulating GM and its metabolites, reducing inflammation and oxidative stress (19). Similarly, *Lactobacillus plantarum* HY7715 supplementation in aged mice improved physical performance and skeletal muscle mass by promoting myoblast differentiation and mitochondrial biogenesis while restoring GM composition (21). *Lactobacillus gasseri* BNR17 has been shown to ameliorate dexamethasone-induced muscle loss in mice and cultured cells by downregulating the protein degradation pathway (myostatin/MuRF1/MAFbx/FoxO3) and upregulating the protein synthesis pathway (IGF-1/Akt/mTOR), thereby improving muscle mass, strength, and endurance (108). *Bifidobacterium animalis* subsp. *lactis* Probio-M8 improves muscle function in aged mice and sarcopenia patients by modulating gut microbial metabolites (e.g., increasing indole-3-lactic acid, acetoacetic acid, and creatine while reducing n-dodecanoyl-L-homoserine lactone), supporting the gut-muscle axis as a therapeutic target for sarcopenia (109). Additionally, *Akkermansia muciniphila* supplementation mitigates sarcopenia in aged SAMP8 mice by suppressing inflammation, preserving gut barrier integrity, and improving the gut microenvironment, thereby enhancing muscle mass and function (110). *Lactobacillus rhamnosus* JY02 alleviates dexamethasone-induced sarcopenia in cells and mice by increasing muscle-enhancement markers and reducing muscle degradation markers and pro-inflammatory cytokines, thereby preventing muscle atrophy and loss of lean mass and strength (111).

Compared to single probiotics, the combined use of multiple probiotics, such as symbiotic bacteria, could be more effective as they can mutually potentiating the effects for the host. For instance, a six-week double-blind trial in 88 non-athletes found that combining exercise training with *Lactobacillus plantarum* PL-02, *Lactococcus lactis* LY-66, or their combination significantly improved muscle strength, endurance, and body composition as well as increasing beneficial GM like *Akkermansia muciniphila*, which demonstrated the synergistic effects of probiotics and exercise on physical performance (112). Another study found that *Lactobacillus paracasei* P62 and *Bifidobacterium bifidum* P61, especially in combination, reduce muscle degradation and inflammation in aged mice by modulating GM and regulating AKT, NF-κB, and FOXO3a signaling pathways (113).

Several factors may influence probiotic efficacy, including baseline GM composition, dietary habits, and lifestyle. Therefore, probiotic selection should be individualized based on patient characteristics. Moreover, some evidence originates from combined interventions cannot be attributed to probiotics alone. For instance, a 2-month randomized trial in 60 sarcopenic elderly patients used a specialized medical food containing omega-3 fatty acids, leucine, and *Lactobacillus paracasei* PS23, which significantly improved appendicular lean mass, muscle strength, and physical performance (114). However, this study does not allow conclusions about the independent effect of the probiotic, as the intervention contained multiple active components. Therefore, clinical applications should consider these limitations (Table 2).

**Table 2:** Probiotics against sarcopenia.

Probiotic strain	Study type	Model	Sample size	Dose	Duration	Co–interventions	Safety	Primary outcomes (muscle–related)	References
*Limosilactobacillus reuteri* ATG–F4	Animal	Aged mice	*n* = 15 (5 mice per group)	4.0 × 10^9^ CFU/mouse	27 days	None	Not reported	↑ muscle mass, ↑ exercise capacity (grip strength, endurance), ↓ inflammatory factors	(105)
*Lactobacillus plantarum TWK10*	Animal	Male ICR mice	*n* = 24 (8 per group)	0, 2.05 × 108, or 1.03 × 10^9^ CFU/kg/day	6 weeks	None	Not reported	↑ grip strength, ↑ swimming endurance, ↑ relative muscle mass, ↑ type I muscle fibers, ↓ fatigue markers	(106)
*Lactobacillus plantarum TWK10*	Human RCT	Elderly with mild frailty	55	High–dose (specific dose NR)	>6 weeks	None	Not reported	↑ muscle strength, ↑ endurance, ↓ sarcopenia/physical frailty symptoms	(101)
*Lactobacillus plantarum PL−02*	Animal	Rats	*n* = 32 (8 mice per group)	2.05 × 10^9^ CFU/kg mouse/day	4 weeks	Resistance training	Not reported	↑ muscle mass, ↑ exercise performance, ↓ fatigue markers (synergistic with training)	(20)
*Lactobacillus plantarum PL−02*	Animal	Male mice	*n* = 40 (10 per group)	0, 2.05 × 10^9^, 4.10 × 10^9^, or 1.03 × 10^1^ CFU/kg/day	4 weeks	None	Not reported	↑ muscle mass, ↑ grip strength, ↑ glycogen storage, ↓ post-exercise fatigue markers (BUN, CK, LD)	(107)
*Lactobacillus casei Shirota*	Animal	Aged SAMP8 mice (16–28 weeks old)	*n* = 24 (6 per group)	1 × 108 or 1 × 10^9^ CFU/mouse/day	12 weeks	None	Not reported	Mitigated age-related muscle decline (modulated GM/metabolites, ↓ inflammation, ↓ oxidative stress, preserved muscle fiber cross-sectional area)	(19)
*Lactobacillus plantarum HY7715*	Animal	Aged Balb/c mice (80 weeks old)	*n* = 24 (6 per group)	0.2 mL/day of 1 × 108−1 × 10^9^ CFU/mL	6 weeks (once daily)	None	Not reported	↑ physical performance, ↑ skeletal muscle mass (myoblast differentiation, mitochondrial biogenesis, restored GM composition)	(21)
*Lactobacillus paracasei PS23 (in medical food)*	Human RCT	Sarcopenic elderly	60	“30 Billion,” freeze dried	2 months	Omega−3 fatty acids, leucine	One adverse event, but not related to the intervention	↑ appendicular lean mass, ↑ muscle strength (handgrip), ↑ physical performance (SPPB), ↓ visceral fat	(114)
*Lactobacillus gasseri BNR17*	Animal + *in vitro*	Dexamethasone–induced muscle loss (BALB/c mice, C2C12 cells)	*n* = 60 (10 per group)	200 μl of PBS (10^6^-10^9^ CFU/mouse/day)	21 days	None	Not reported	↑ muscle mass (gastrocnemius, quadriceps), ↑ grip strength, ↑ treadmill running time, ↑ rotarod retention time, ↑ lean body mass (DXA), ↓ fat mass (↓ degradation pathway, ↑ synthesis pathway)	(108)
*Bifidobacterium animalis subsp. lactis Probio–M8*	Animal + human	Aged mice + sarcopenia patient	For mice, *n* = 12 (6 per group); For patients, *n* = 43	For mice, 5 × 10^8^ CFU every 2 days; For patients, 1.8 × 10^8^ CFU	For mice, 28 days; For patient, 60 days	None	Not reported	↑ muscle function (modulation of metabolites: ↑ indole-3-lactic acid, acetoacetic acid, creatine; ↓*n*-dodecanoyl-L-homoserine lactone)	(109)
*Akkermansia muciniphila*	Animal	Aged SAMP8 mice (7 months old)	*n* = 17	1 × 10^8^ CFU per mouse, five times per week	3 months	None	Not reported	↑ grip strength, ↑ skeletal muscle mass, suppressed cellular senescence, improved protein degradation/synthesis balance, ↑ total ATP, ↑ mitochondrial biogenesis, preserved gut barrier integrity, ↓ inflammatory cytokines	(110)
*Lactobacillus rhamnosus JY02*	Animal + *in vitro*	Dexamethasone–induced sarcopenia (mice, C2C12 cells)	Not reported	1 × 10^8^ CFU/mouse, 0.1 ml/mouse, daily	5 weeks	None	Not reported	Prevented muscle atrophy, loss of lean mass and muscle strength (↑ muscle-enhancement markers MHC Iβ, MHC IIα, Myo-D; ↓ degradation markers MuRF1, atrogin-1; ↓ pro-inflammatory cytokines IL-6, IFN-γ; ↑ anti-inflammatory IL-10)	(111)
*Lactobacillus plantarum PL−02 + Lactococcus lactis LY−66*	Human RCT	Non–athletes	*n* = 88	7.5 × 10^9^ CFU/sachet, 2 sachets/day	6 weeks	Exercise training	Not reported	↑ muscle strength (1-RM squat), ↑ endurance (countermovement jump), ↑ body composition, ↑ beneficial GM (Akkermansia muciniphila)	(112)
*Lactobacillus paracasei P62 + Bifidobacterium bifidum P61*	Animal	Aged mice	*n* = 6 (6 per group)	1 × 10^9^ CFU/mouse, 6 days per week	8 weeks	None	Not reported	↓ muscle degradation (increased grip strength, treadmill running time/distance, muscle weight), ↓ inflammation (modulated GM, AKT/NF-κB/FOXO3a signaling), combination most effective	(113)

BUN, blood urea nitrogen; CK, creatine kinase; DXA, dual–energy X–ray absorptiometry; GM, gut microbiota; ICR, Institute of Cancer Research; LD, lactate dehydrogenase; NR, not reported (information not available in the cited reference); RCT, randomized controlled trial; SPPB, Short Physical Performance Battery. Safety data were not systematically reported in most included studies; future trials should routinely document adverse events. References correspond to the numbering in the manuscript.

#### Dietary intervention

Dietary intervention is a crucial method for regulating GM and alleviating sarcopenia. To avoid over-attributing general nutritional benefits to the gut-muscle axis, we first distinguish between direct nutritional effects on muscle and GM-mediated effects. A study on NHANES data from 2007 to 2018 found that the higher the gut microbiota dietary index (DI-GM) score, the lower the risk of sarcopenia (115). This effect is partially mediated by biological age indicators, especially PhenoAge, which accounts for 30.6%. A reasonable dietary structure can maintain the balance of GM, promote the growth of beneficial bacteria, and reduce the proliferation of harmful bacteria. Increasing dietary fiber intake can provide prebiotic substrates, promote the fermentation of beneficial bacteria in the intestine to produce SCFAs, and have a positive impact on muscle health (116, 117). For example, the Mediterranean diet, which is rich in vegetables, fruits, whole grains, olive oil, etc., possesses anti-inflammatory and antioxidant properties and is associated with beneficial changes in GM and a lower incidence of sarcopenia (118, 119). Moreover, adequate intake of protein, vitamin D, antioxidants, and long-chain polyunsaturated fatty acids (LCFAs) has been shown to directly help prevent sarcopenia through nutrient-muscle interactions, without primarily relying on GM modulation (120).

Furthermore, protein intake is crucial for maintaining muscle mass and function. Adequate protein intake, especially high-quality protein, can provide the amino acids necessary for muscle synthesis, helping to prevent and improve sarcopenia (121). Some studies have found that combining specific dietary patterns, such as increasing the proportion of plant protein, with guaranteed protein intake may yield synergistic benefits for GM and muscle health. Additionally, certain food components, such as polyphenols, have the effects of regulating GM, reducing inflammation, and protecting muscles; in contrast, omega-3 fatty acids act mainly via direct anti-inflammatory pathways rather than GM modulation (122, 123). High-protein diet (HPD) can significantly alter the composition of GM, increasing the abundance of certain bacteria related to protein metabolism, such as Bacteroides and Firmicutes, which can more effectively decompose proteins and produce SCFAs (124, 125). However, the primary anabolic effect of HPD on muscle is attributed to direct amino acid supply; the GM-mediated contribution remains secondary. Moreover, excessive protein intake could stimulate insulin and glucagon secretion and impair insulin action, as well as increase the potential risk of prediabetes and type 2 diabetes mellitus (T2DM) (126). Therefore, when following a high-protein diet, it is necessary to consider its potential impact on GM and optimize gut health by reasonably combining other components such as dietary fiber.

Dietary fiber is a crucial component for regulating GM. Various types of dietary fiber, such as pectin, oligofructose, lactulose, and resistant starch, can selectively promote the growth of beneficial bacteria and increase microbial diversity in the gut, thereby improving intestinal health and metabolic function (127, 128). For instance, fermentation products of dietary fiber, such as SCFAs, have been found to be closely related to muscle metabolism and function. SCFAs exert their influence on mucosal homeostasis through multiple mechanisms, such as maintaining the intestinal barrier, increasing the number and activity of regulatory T-cells, and suppressing the expression of various inflammatory cytokines (7). Studies have shown that increasing dietary fiber intake can elevate the levels of SCFAs, which in turn positively impact muscle mass (129). Therefore, a reasonable intake of dietary fiber can not only improve the composition of GM but also support muscle health by enhancing metabolic status, providing a new dietary intervention strategy for the prevention and treatment of sarcopenia.

#### FMT

Accumulating evidence from preclinical studies has highlighted the therapeutic potential of FMT. For instance, one study demonstrated that FMT from sarcopenic donors could induce muscle loss in mice, while *Lacticaseibacillus rhamnosus* and *Faecalibacterium prausnitzii* supplementation could alleviate age-related muscle disorders by enhancing mitochondrial function via TCA cycle activation (130). Similarly, FMT from hemodialysis patients with sarcopenia into mice induced muscle dysfunction and reduced Akkermansia abundance, demonstrating that GM disruption may directly contribute to sarcopenia in this population (131). Extending these findings, another study showed that transplanting GM from CKD mice into healthy germ-free mice leads to reduced muscle mass, impaired insulin signaling, mitochondrial dysfunction, systemic inflammation, and elevated uremic solutes (132). In contrast, FMT from young donors could alleviate sarcopenia in aged rats by reshaping GM and metabolites, maintaining gut barrier integrity, and improving muscle mitochondrial function (47).

Translational evidence in humans remains limited. A retrospective study involving 172 sarcopenia patients reported that FMT combined with resistance training significantly improved muscle mass, function, and inflammatory markers compared to resistance training alone, with complete remission rates of 46.7% versus 32.4% (4). However, due to the retrospective design, these findings should be interpreted with caution. Several prospective trials are ongoing; for example, a double-blind, randomized placebo-controlled trial protocol has been published evaluating oral FMT from young, physically active donors in older adults (aged 65–84) could improve muscle health, cognitive function, and metabolic status (133). Collectively, these studies highlight the great potential of FMT in the treatment of sarcopenia.

Importantly, FMT remains an experimental approach for sarcopenia and is not yet an established treatment. Safety and regulatory considerations are critical, especially in older adults and patients with CKD or immunocompromised conditions. Key issues include donor screening for pathogens, risk of transmitting multidrug-resistant organisms or antimicrobial resistance genes, potential for long-term disruption of the recipient's GM, route of administration (e.g., oral capsules, retention enema or colonoscopy), and ethical oversight. Regulatory frameworks (e.g., FDA and EMA requirements for investigational FMT products) must be adhered to. Therefore, future randomized controlled trials should prioritize both efficacy and rigorous adverse event monitoring.

### Critical appraisal of current evidence

The strongest evidence comes from: (1) Animal FMT studies showing that GM transfer reproduces muscle phenotypes; (2) SCFAs/mTOR signaling, with butyrate promoting protein synthesis and suppressing NF-κB inflammation; and (3) Mendelian randomization studies providing genetic support for causal effects of specific taxa (e.g., *Bifidobacterium, Butyricimonas*) and butyrate synthesis on muscle traits. In contrast, bile acids, tryptophan metabolism, AMPK, and mitochondrial pathways remain speculative, as most supporting data are from animal models or indirect inference without human mechanistic validation.

Conflicting evidence is most apparent for BCAAs. Observational studies report positive associations between circulating BCAAs and muscle mass, yet an RCT in cirrhosis found no benefit of BCAA supplementation over whey protein. These findings are not incompatible: circulating levels (endogenous), exogenous supplementation, and catabolic capacity are distinct concepts. Elevated BCAAs may signal impaired breakdown rather than sufficient availability. Thus, BCAA efficacy likely depends on disease context, metabolic status, and individual utilization efficiency, and findings should not be generalized across populations without caution.

Major confounders, including age, diet, medications (PPIs, antibiotics), comorbidities (CKD, diabetes, heart failure), and diagnostic criteria (EWGSOP2 vs. AWGS), are not often fully adjusted and may explain substantial GM variability across studies.

Future trials should measure: GM composition (metagenomics), fecal/circulating SCFAs, inflammatory markers (IL-6, TNF-α, CRP), muscle protein synthesis rate, BCAA catabolic capacity, and harmonized sarcopenia outcomes using standardized criteria (Table 3).

**Table 3:** Evidence level of key mechanisms and interventions linking gut microbiota to sarcopenia.

Theme	*In vitro*/Animal models	Human observational studies	Clinical intervention
Inflammation	GM dysbiosis increases LPS translocation, activates NF–κB, elevates TNF–α/IL−6, induces muscle atrophy via mTOR/FoxO pathways (66–69, 92)	Higher circulating IL−6, TNF–α associated with lower muscle mass/strength (24); reduced GM diversity correlates with inflammaging (66, 68)	Probiotics (*L. rhamnosus, L. plantarum*) reduce inflammatory markers and improve muscle outcomes in RCTs (101, 107, 111)
SCFAs	Butyrate/acetate/propionate activate AMPK/mTOR, improve mitochondrial function, reduce autophagy in aged sarcopenic mice (5, 17, 89, 91)	Fecal butyrate positively correlated with skeletal muscle index in older adults (55); butyrate–producing bacteria (*F. prausnitzii*) enriched in healthy muscle (90)	Fiber–rich diet increases SCFAs and improves muscle health (116, 129); SCFA supplementation not yet tested in human sarcopenia RCTs
BCAAs	*P. copri* supplementation increases blood BCAAs and muscle mass in mice (84); impaired BCAA catabolism (BT2) protects against sarcopenia (85); *P. merdae* BCAAs metabolites delay aging (87)	Circulating BCAAs positively associated with muscle mass/strength; valine linked to 47% lower sarcopenia risk (86); lower *P. copri* in sarcopenic individuals (84)	12–month RCT in cirrhosis: no added benefit of BCAA supplementation vs whey protein (88); effects likely context–dependent
Bile acids	Bile acid signaling via FXR/TGR5 regulates muscle protein turnover and autophagy (95); indoxyl sulfate induces myostatin/atrogin-1 in C2C12 cells and mice (97)	Altered bile acid profiles in sarcopenic patients with cirrhosis (41); FXR/TGR5 dysregulation linked to sarcopenia (95)	No direct RCT; dietary fibers and probiotics modulate bile acid metabolism indirectly
Tryptophan metabolism	GM shifts tryptophan to kynurenine/serotonin/indole, promoting gut and muscle inflammation in animal models (96)	Indoxyl sulfate (uremic toxin) elevated in CKD–associated sarcopenia (97); tryptophan metabolites altered in frail elderly (96)	No RCT targeting tryptophan pathway for sarcopenia
Probiotics	Multiple strains (*L. plantarum* TWK10, PL−02, HY7715; *L. casei* Shirota; *L. gasseri* BNR17; *B. animalis* Probio–M8; *A. muciniphila*; *L. rhamnosus* JY02; *L. paracasei* P62 + *B. bifidum* P61) increase muscle mass, strength, endurance in aged/atrophied mice (19, 20, 103, 105–111, 113)	Cross–sectional studies: beneficial bacteria (*Bifidobacterium, Lactobacillus*) lower in sarcopenic individuals (45, 47)	Several RCTs show probiotics (e.g., *L. plantarum* TWK10, *L. paracasei* PS23, *B. animalis* Probio–M8) improve muscle mass/strength in elderly/frail adults (101, 109, 114); meta–analysis supports positive effect (52)
Dietary interventions	High–protein diet alters GM composition and SCFA production in rats (124); fiber increases butyrate and protects against muscle atrophy in mice (129); polyphenols regulate GM and reduce inflammation (122)	Mediterranean diet, higher dietary fiber intake associated with lower sarcopenia risk (118, 119); DI–GM score inversely related to sarcopenia (115)	RCTs show protein supplementation ± exercise improves muscle outcomes; fiber–rich diet RCTs are ongoing
FMT	FMT from sarcopenic donors induces muscle loss in mice (130, 131); FMT from young donors alleviates sarcopenia in aged rats (47)	Retrospective study (*n* = 172): FMT + resistance training improved muscle mass/function vs training alone (4); case reports limited	One RCT protocol (ARMOR study) ongoing; no published RCT results yet (133)

BCAA, branched-chain amino acid; CKD, chronic kidney disease; DI-GM, dietary index for gut microbiota; FMT, fecal microbiota transplantation; GM, gut microbiota; LPS, lipopolysaccharide; RCT, randomized controlled trial; SCFA, short–chain fatty acid. References correspond to the numbering in the manuscript. Mendelian randomization evidence is only available for SCFAs (butyrate).

## Discussion

**Figure 2:**
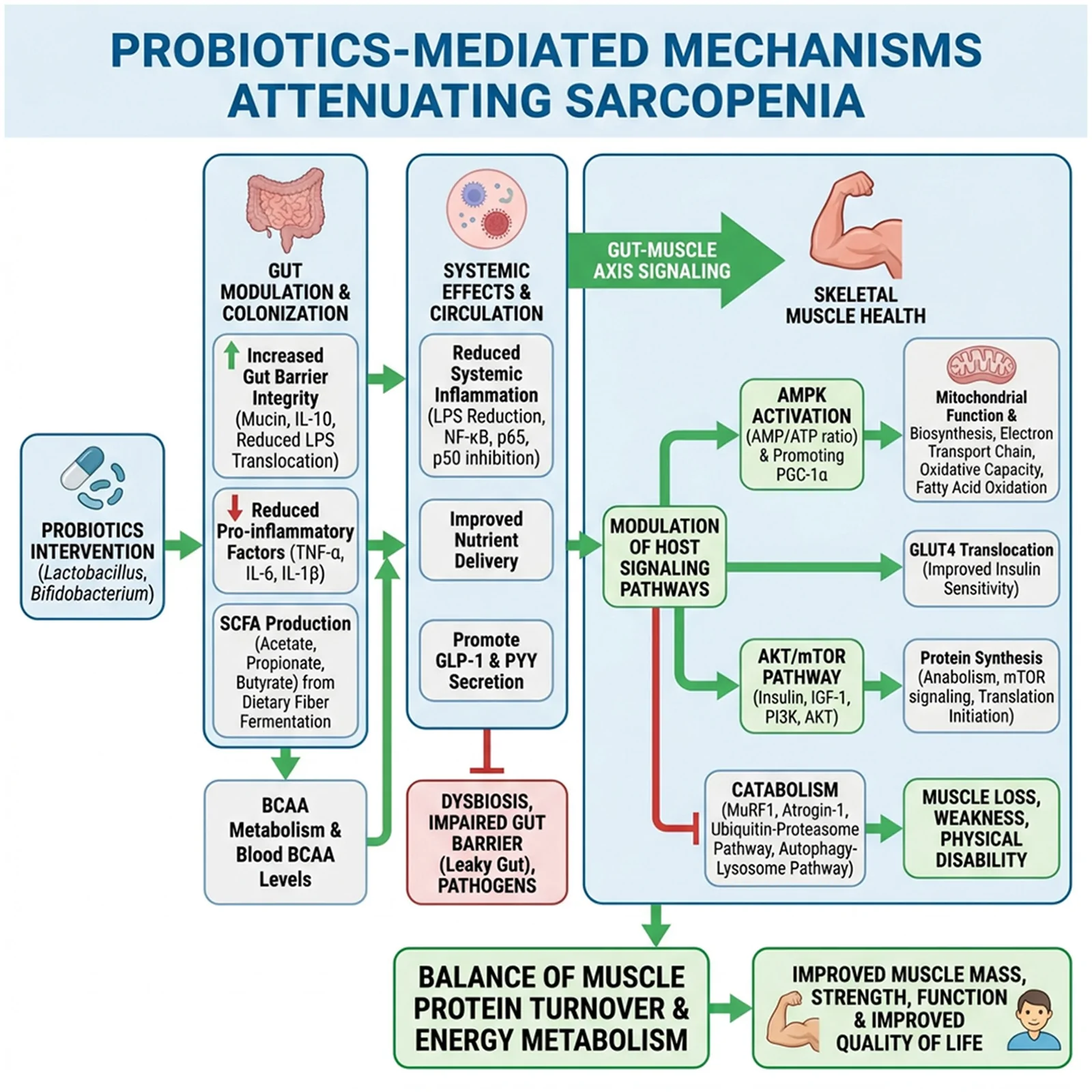
Probiotics improve gut homeostasis by enhancing intestinal barrier integrity (e.g., mucin production, IL-10), reducing LPS translocation, and lowering pro-inflammatory cytokines (TNF-α, IL-6, IL-1β). The fermentation of dietary fiber produces short-chain fatty acids (SCFAs; acetate, propionate, butyrate), which regulate branched-chain amino acid (BCAA) metabolism and systemic BCAA levels. These changes lead to reduced systemic inflammation (NF-κB p65/p50 inhibition), improved nutrient delivery, and enhanced secretion of GLP-1 and PYY. Through gut-muscle axis signaling, probiotics activate AMPK (via AMP/ATP ratio) and promote PGC-1α, while modulating host pathways including AKT/mTOR signaling (insulin, IGF-1, PI3K, AKT) and suppressing catabolic pathways (MuRF1, Atrogin-1, ubiquitin-proteasome, autophagy-lysosome). Additionally, they enhance GLUT4 translocation and insulin sensitivity, which promote protein synthesis. Collectively, these mechanisms rebalance muscle protein turnover and energy metabolism, ultimately improving muscle mass, strength, function, and quality of life.

**Figure 3:**
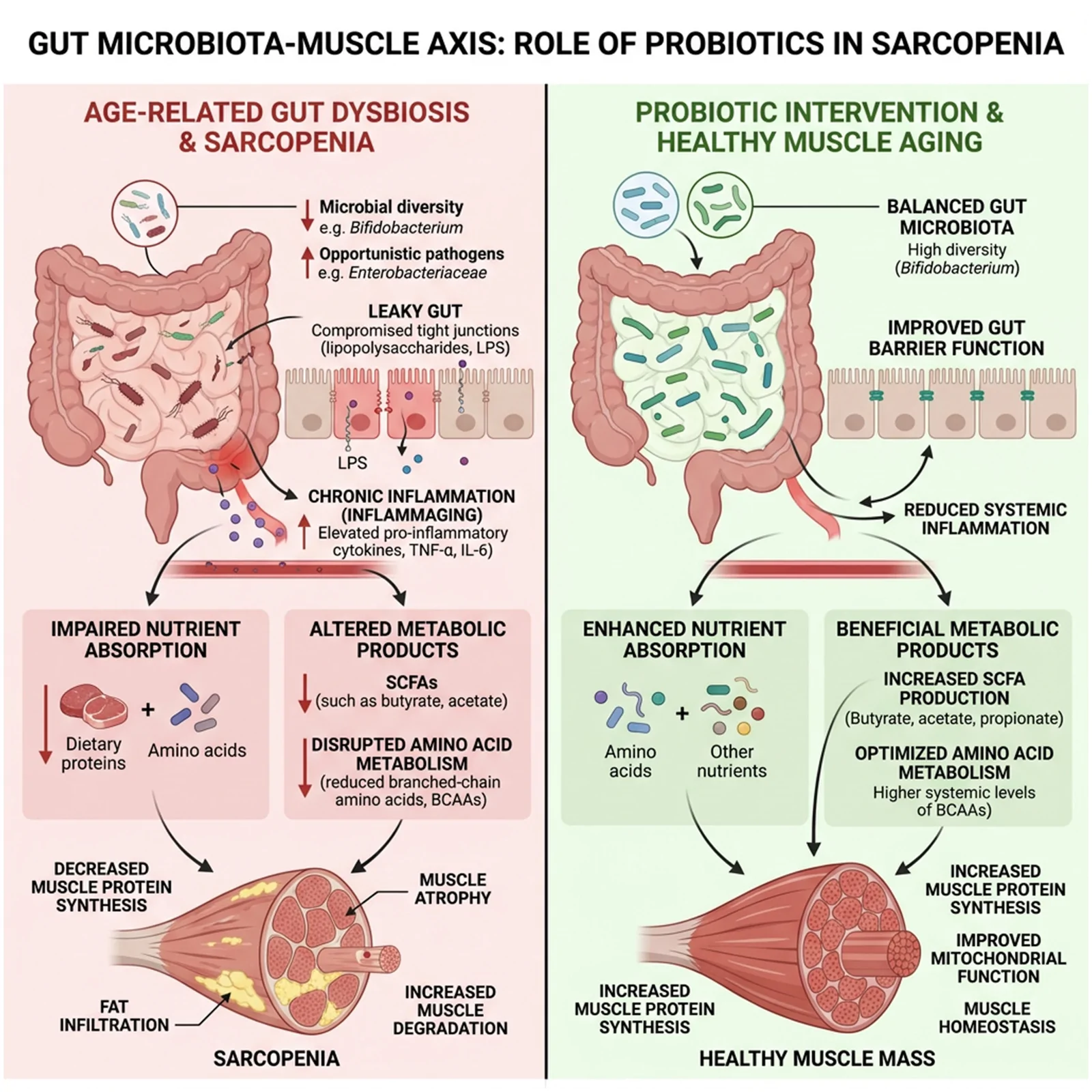
Pivotal role of GM in sarcopenia. The schematic contrasts age-related gut dysbiosis–associated sarcopenia **(left)** with probiotic-supported healthy muscle aging **(right)**. During aging, reduced microbial diversity (e.g., decreased Bifidobacterium) and expansion of opportunistic pathogens (e.g., Enterobacteriaceae) are associated with impaired gut barrier integrity (“leaky gut”) via compromised tight junctions, facilitating translocation of lipopolysaccharides (LPS) and promoting chronic low-grade systemic inflammation (inflammaging) characterized by elevated pro-inflammatory cytokines (e.g., TNF-α, IL-6). These changes contribute to impaired nutrient absorption and altered microbial metabolic outputs, including reduced short-chain fatty acids (SCFAs; e.g., butyrate, acetate) and disrupted amino acid metabolism with lower branched-chain amino acids (BCAAs), ultimately decreasing muscle protein synthesis and promoting muscle atrophy, fat infiltration, and increased muscle degradation, culminating in sarcopenia. In contrast, probiotic intervention is depicted as supporting a balanced, diverse GM, improving intestinal barrier function and reducing systemic inflammation. These effects are associated with enhanced nutrient absorption and beneficial metabolic profiles, including increased SCFA production (butyrate, acetate, propionate) and optimized amino acid metabolism (higher systemic BCAAs), which together promote muscle protein synthesis, improve mitochondrial function, maintain muscle homeostasis, and preserve healthy muscle mass.

In contemporary medical research, the GM is increasingly recognized as a crucial physiological system, and its role in host health and disease progression is now extensively studied. Several reports have showed that dysbiosis of GM closely correlates with sarcopenia. GM may contribute to sarcopenia via the regulation of various pathways including muscle metabolism, inflammatory response and nutrient absorption. Therefore, it is important to investigate the effect of GM on energy metabolism in muscle and inflammation. The composition and metabolites of the GM can influence muscle synthesis and degradation. For instance, SCFAs produced by probiotics can promote muscle protein synthesis and reduce inflammation, thereby affecting muscle quality and function (90). In contrast, an increase in harmful GM is correlated with enhanced inflammation and muscle wasting. Additionally, GM participates in nutrient absorption, influencing the overall nutritional status of the host and indirectly contributing to muscle health. Therefore, dysbiosis in GM might be an important mechanism in the development of sarcopenia (Figure 2, Figure 3).

Interventions targeting GM are gradually emerging as an important research direction for the prevention and treatment of sarcopenia. Studies have shown that restoring a healthy GM through the use of probiotics, prebiotics, or FMT may have a positive effect on improving muscle health. For example, some research has found that supplementing with probiotics can enhance muscle mass and function, providing new possibilities for the treatment of sarcopenia. In addition, dietary interventions, such as high-protein diets and fiber-rich diets, are also believed to help optimize the structure of GM, thereby indirectly promoting muscle health. The evidence level of key mechanisms and interventions linking GM to sarcopenia is shown in Table 4.

**Table 4:** Critical appraisal of current evidence in gut microbiota–sarcopenia research.

Aspect	Summary
Strongest evidence	Animal FMT; SCFAs/mTOR; MR studies
Speculative	Bile acids; tryptophan; AMPK; mitochondrial pathways
Conflicting	BCAAs (observational positive vs. RCT null)—reflects distinct concepts: circulating levels vs. supplementation vs. catabolic capacity
Key confounders	Age, diet, medications, comorbidities, diagnostic criteria
Future measures	Metagenomics; SCFAs; inflammatory markers; muscle protein synthesis; BCAA catabolism; harmonized outcomes

Sarcopenia is a highly heterogeneous disease that can be accompanied by different disease states, such as chronic cardiovascular disease, kidney disease, obesity. These accompanying disease states may affect changes in GM (134, 135). The interactions between GM and other metabolic diseases also warrant attention. These interactions may further promote the development of sarcopenia by affecting metabolic pathways, inflammatory states, and muscle adaptability. For instance, dysbiosis of GM may be associated with metabolic syndrome, which itself is closely related to the decline in muscle mass. Therefore, future research is necessary to delve into how GM indirectly affects the pathological mechanisms of sarcopenia by regulating other metabolic diseases, thereby providing a theoretical basis for developing new intervention strategies.

Individualized treatment strategies are one of the important directions for future research. Due to significant differences in the composition and responsiveness of GM among individuals, developing individualized intervention plans may be more effective in improving sarcopenia symptoms. With the help of genomics and metabolomics technologies, researchers can identify specific GM markers associated with sarcopenia and design targeted nutritional or therapeutic plans accordingly (136, 137). This individualized approach not only helps to enhance treatment efficacy but also reduces unnecessary side effects, providing patients with more precise intervention options.

Future research should focus on the following key directions. Firstly, it is necessary to identify specific GM closely related to the development of sarcopenia, and deeply reveal the complex mechanism between GM and sarcopenia, laying the foundation for clinical intervention. Secondly, through designing rigorous clinical trials, the actual effects of probiotics and other microbial treatments in patients with sarcopenia should be evaluated to verify their clinical application value. In addition, with the development of multi-omics technology, integrating data from genomics, metabolomics, and other fields for systematic analysis is expected to provide a more comprehensive perspective on understanding the relationship between GM and sarcopenia, promoting the in-depth development of related research.

In summary, GM plays a crucial role in the pathogenesis of sarcopenia, and the exploration of its influencing mechanisms will offer novel insights and approaches for the prevention and treatment of sarcopenia. By integrating various research findings and balancing diverse viewpoints and discoveries, we can establish a more robust theoretical foundation for future studies, aiming to enhance the quality of life for sarcopenia patients and facilitate a healthy aging process.
